# *Notice to Readers:* Ongoing Analysis of Suicide Rates Data by Occupational Group from Results Reported in *MMWR*

**DOI:** 10.15585/mmwr.mm6725a7

**Published:** 2018-06-29

Recently, *MMWR* Editors were informed by the authors of “Suicide Rates by Occupational Group — 17 States, 2012” (*1*) that some results and conclusions might be inaccurate as a result of coding errors for certain occupational groups. The authors are undertaking a thorough reanalysis of the data. This notice is to alert readers about the coding errors while the reanalysis is conducted to assess the validity of results and conclusions in the publication.
